# Intelligence and the Individuals with Disabilities Education Act

**DOI:** 10.3390/jintelligence7040024

**Published:** 2019-11-21

**Authors:** Tomoe Kanaya

**Affiliations:** Department of Psychological Science, Claremont McKenna College, Claremont, CA 91711, USA; tkanaya@cmc.edu

**Keywords:** IDEA, special education, group differences in intelligence, Larry P. v Riles

## Abstract

One of the stated purposes of this Special Issue is to “discuss when and why intelligence has disappeared” in education. In this paper, I argue that intelligence is still heavily involved in public education in the United States due to the Individuals with Disabilities Education Act. Moreover, due to several factors, including high-profile court cases, intelligence tests are legally used in an inconsistent manner in special education decision-making throughout the U.S. These cases illustrate the complex issues surrounding the psychometric properties of intelligence tests, historical conflicts surrounding racial equity, differences in federal versus state policies, and methodological concerns surrounding special education policies are discussed.

## 1. A Brief History of Compulsory Education, the Elementary and Secondary Education Act, and the Individuals with Disabilities Education Act

Compulsory, elementary education was an established practice in parts of the original 13 colonies before the Revolutionary War (i.e., Massachusetts in 1647) [1]. After independence from England, Massachusetts was the first state to pass a compulsory, elementary education law in 1852 [2]. In 1892, the National Educational Association recommended the extension of compulsory education to twelve years, and by 1918 (over 50 years after the civil war ended in 1865), every state had signed compulsory education laws [1,3]. During this time, Plessy v Ferguson (1896) created the “separate but equal” standard, which legalized racial segregation in public spaces as long as segregated groups received equal treatment [4]. In practice, this standard often led to discriminatory practices and gross inequalities, leading to the landmark case, Brown v Board of Education of Topeka (1954) [5]. In Brown, the courts ruled that separate educational facilities violated the 14th Amendment, which guarantees equal protection and eliminated segregation. 

While children had access to integrated schools, the educational quality provided to them still varied greatly. Children living in poverty, in particular, were experiencing lower quality educational experiences and the worst academic outcomes [6]. Therefore, the Elementary and Secondary Education Act of 1965 was passed to provide federal funding to low-income and underperforming schools [7]. Now called Every Student Succeeds Act (ESSA), ESSA continues to implement specific educational standards at a federal level, such as all students who graduate high school are required to be ‘college and career ready’ [8]. However, the system of education, such as developing grade-based curriculum, is created and mandated by individual states under the 10th Amendment. 

While the educational rights of the poor and racial minorities were now protected, children with disabilities were still struggling to receive the same educational opportunities as their non-disabled counterparts. This led to the passing of Education for All Handicapped Children Act, guaranteeing free and appropriate educational services to children with disabilities [9]. Renamed the Individuals with Disabilities Education Act (IDEA) in 1990, IDEA requires a measure of “general intellectual ability” to determine if a student is eligible for one of the 13 IDEA diagnoses: Autism, Blindness, Deafness, Emotional Disturbance (ED), Hearing Impairment, Intellectual Disability (ID), Multiple Disabilities, Orthopedic Impairment, Other Health Impaired, Specific Learning Disability (LD), Speech, Traumatic Brain Injury, and Visual Impairment [10]. 

These policies reflect the difficulties in creating an educational system that would serve, as envisioned by Horace Mann [11], “a great equalizer in of the conditions of man—the balance wheel of the social machinery” (pg. 59). Pointing to the seminal role of his teachers on his own ascent, President Lyndon B. Johnson called education the “only valid passport from poverty” when signing the ESEA [12]. And yet, school children across the country continue to experience disparate levels of educational opportunities and access due, in part, to the historically politicized context surround race and poverty, state-level variability protected under ESSA, funding differences between districts [13], and socioeconomic factors at the individual level [14]. Similarly, while IDEA is to ensure that children with disabilities are served with equal educational opportunities as their non-disabled counterparts, it is difficult to determine how to do this and if it is effective. Several high-profile litigations that the focus on the role of intelligence measures in IDEA assessments and recent research findings on the efficacy of IDEA services highlight this difficulty and are outlined briefly below.

## 2. Intelligence and IDEA in the Courts

Two significant court cases quickly highlighted the difficulties of using IQ in education and special education placement decisions. Hobson v Hansen (1967) questioned the use of IQ to track students in Washington D.C., arguing this practice led to an over-representation of African American students in lower tracks compared to their White counterparts, violating the 14th Amendment [15]. Similarly, Larry P. v Riles (1979) argued IQ tests were used to diagnose African American students with Intellectual Disability (ID) for discriminatory purposes, leading to an overrepresentation of African Americans in special education classrooms in Northern California [16]. 

In both cases, the plaintiffs argued the IQ test available at the time was an inappropriate assessment to use on African Americans because it was standardized on an all-White sample. Moreover, they provided evidence that IQ performance was used as the sole assessment for these placements, which was in violation of educational policy and portrayed discriminatory intent. The courts agreed and emphasized the need for schools to base educational placement decisions on students’ individual, educational needs, nor could they rely on assessment data that served as proxies for race, ethnicity, and socioeconomic factors. In addition, both courts mandated the elimination of tracking in D.C. and over-representation of minorities in special education in California. 

Similarly, Diana v California State Board of Education (1970) questioned the use of IQ tests on nine Mexican-American students whose primary language was Spanish and later placed in ID classroom [17]. The plaintiffs argued the students’ low performances were not due to a cognitive deficit, but to their language barrier that made it difficult for them to understand the test questions. The courts agreed and ruled that all IDEA evaluations in California must include nonverbal assessments or assessments in the students’ primary language. 

This perspective, however, was not upheld in all cases. Specifically, in Parents in Action on Special Education (PASE) v Hannon (1980), the Chicago judge disagreed with the plaintiffs, who argued the overrepresentation of African American students in ID classrooms were a result of discriminatory practices that relied too heavily on IQ tests [18]. The judge examined specific test questions and determined the test itself was not discriminatory against race, and when used in conjunction with other mandated assessments and procedures, would not lead to racially biased ID diagnoses. While this ruling may seem contradictory given previous rulings, addition context might help: The ID and lower-track placements of African Americans in Larry P. and Hobson were problematic because the schools relied almost entirely on the students’ IQ performance and did not include other assessments (such as behavioral data) in evaluations, which is required under IDEA. Furthermore, the courts found the respective classrooms were providing inferior educational experiences compared to the experiences provided to the non-ID and upper track students. More specifically, the content of the ID curriculum was determined to be a “dead end” in Larry P. [19]. This was also the situation, in Lora v Board of Education of New York (1977), African American and Hispanic students were disproportionately diagnosed with ED and placed into separate schools where services were determined to be “inadequate” [20]. Therefore, the same discriminatory intent and issues of equal protection were not found in *PASE*. 

The appropriateness of IQ in determining ID placements of African Americans in Georgia was also argued in Georgia State Conference of Branches of NAACP v State of Georgia (1985) [21]. While the plaintiffs were able to show procedural irregularities, including missing documentation and misuse of the IQ guidelines, they were not able to show these irregularities were used in a discriminatory manner based on race. Rather, African American and White students were experiencing them. Finally, the court found the ID curriculum provided a tailored educational experience that was beneficial to students with lower cognitive ability [22], making notable improvements from the substandard experiences experienced by the plaintiffs in Hobsen and Larry P.

While litigations surrounding the ID diagnosis have focused on reducing the number of students receiving services, cases surrounding the LD diagnosis often have the opposite goal. The ID diagnosis requires sub-average intellectual functioning while the LD diagnosis requires an at-least-average IQ [10]. Furthermore, a discrepancy between an individual’s IQ and achievement (where a student’s IQ is higher than their achievement test score) is a commonly used criterion for the LD diagnosis. The 2019, high-profile college admissions scandal, FBI’s Operation Varsity Blues, show that high-income parents are willing to commit crimes so their children can receive LD services, including extra time on and distraction-free settings for standardized tests [23]. In the original 1979 Larry P. ruling, the judge prohibited the use of IQ tests on ID diagnoses for African Americans. In 1986, he expanded the ban for all IDEA evaluations on African Americans. This ruling, however, was vacated in Crawford v Honing (1994), when African American plaintiffs in California plaintiffs argued the IQ ban led to discriminatory practices that prevented them from qualifying for and receiving LD services due to their race [24]. While these cases are a limited sample of the numerous litigations surrounding intelligence and IDEA, they represent the complicated and dynamic controversies surrounding intelligence and education. 

## 3. Methodological Difficulties in Examining Intelligence and IDEA 

Since their first edition, subsequent IQ norms have included other races/ethnicities in the standardization samples to address concerns of generalizability. For example, the current Wechsler Intelligence Scale for Children-V [25] was standardized using a sample of children who identified as African American, Asian, Hispanic, and “Other,” as well as children with a wide range of parental education. Even with generalizable standardization samples, many groups (including African Americans, Hispanics, and low-income individuals) perform on average, 8–10 points lower than their White and higher-income counterparts on current IQ norms [26]. There is not a consensus among researchers regarding the cause of these group differences in intelligence [27]. Studies consistently reveal IQ tests have predictive validity on positive outcomes across all races, including academic achievement and occupational attainment, validating their use for all groups [28]. In addition, differential item functioning analyses have not shown group differences on individual items on intelligence tests [28]. Others argue that nationally representative samples do not adequately address the multicultural contexts that are often present in real-life diagnostic situations and will lead to erroneous diagnoses and treatment plans [29,30]. 

These group differences make it difficult to determine the appropriate level of representation in special education. For example, members of low-performing groups will have a higher likelihood of receiving lower scores, which, in turn, lead to an increased likelihood of ID diagnoses and decreased likelihood of LD diagnoses [31,32]. Morgan and colleagues [33,34] have found that minority children are *under*represented in special education classrooms compared to their White counterparts with similar profiles in academic achievement, income, and language status. Others, however, argue these data and analytical approaches do not adequately capture important factors and interdisciplinary nuances of IDEA and minority children with disabilities [35,36]. Finally, given educational quality and resources can vary widely across the country, it is debatable if the benefits of receiving services outweigh issues such as stigmatization that arise from being labeled with a diagnosis for students [32].

In the Georgia case, one of the expert witnesses recommended conducting a study where students with ID are randomly assigned to different levels of IDEA services, including regular education [22]. This recommendation was dismissed for several reasons, including concerns that denying services to students who have been diagnosed with ID could be harmful [22]. These concerns, however, illustrate another layer of complexity when examining the relationship between intelligence and IDEA: 1) there is controversy regarding the efficacy receiving IDEA services, 2) determining the efficacy of IDEA requires random assignment of services [37], and 3) if a student qualifies for services, it is illegal (and potentially unethical) to deny them. Because of these concerns, some educational researchers have utilized propensity score analyses, a methodology that can “mimic” some of the properties of random assignment on observational data, for review, see [38]. 

Specifically, using propensity score analyses on the Early Childhood Longitudinal Study, Kindergarten Class of 1998–1999 dataset, Morgan, Frisco, Farkas, and Hibel [39] concluded that “special education services…during their elementary years may not be of sufficient strength to prevent a subsequent lack of basic skills proficiency” (pg. 208). When examining the impact of preschool IDEA services with the same methodology using the Early Childhood Study-Birth Cohort dataset, Sullivan and Field’s [40] results suggest, “children with delays would indeed demonstrate higher kindergarten academic skills on average if they had not received preschool special education services” (pg. 256). Finally, using the National Longitudinal Survey of Youth and Child and Young Adult datasets, Kanaya, Wai, and Miranda [41] conducted a one-to-one matched propensity score analyses to examine the impact of receiving IDEA services on adulthood outcomes, including economic, social, and health variables. Their results showed individuals who “received special education services did not differ on adulthood outcomes compared to individuals with the same likelihood of receiving services who did not receive services” (pg. 10) on most of their measured outcomes. 

While these results show a consistent pattern across multiple nationally representative datasets that span several cohorts, propensity score analyses are not synonymous with random assignment and causal inferences may be premature. Furthermore, analyses on national datasets are restricted to variables that serve a wide array of research disciplines. Datasets consisting of measures that are tailor-made to the specific contexts surrounding IDEA and children with disabilities may yield different results. Finally, it is difficult to identify and to prioritize the hypotheses required for examining IDEA. 

Specifically, the stated purpose of IDEA is to provide free and appropriate public education services to children who are determined to experience academic difficulties due to their disability. While there are requirements regarding the diagnostic process and treatment options provided (e.g., using the least restrictive environment, evidence of response to treatment) [10], there is not a requirement that individuals experience the same achievements as their regular education counterparts. This was specifically stated by Supreme Court Justice Rehnquist in Board of Education of the Hendrick Hudson Board Central School District v Rowley (1982) who delivered the majority ruling that IDEA services must meet the state’s educational standards but are not required to provide an opportunity to allow each child to achieve his or her “full potential” [42]. Therefore, while numerous studies have reliably shown the positive relationship between years of education and intelligence [43,44], similar expectations are not legally required for children served under IDEA.

## 4. Conclusions 

In the early 1900s, the French government commissioned Alfred Binet and Theodore Simon to develop a measure that could determine which children required additional assistance to complete their compulsory education [45]. Binet was adamant that his measure should not be used as a sole indicator of a child’s intellectual potential and that intelligence can change and develop over the lifespan. His measure was the impetus for the Stanford-Binet, which, along with the Wechsler Intelligence Scale for Children, are the most commonly used IQ measures in the United States, where school children with disabilities are one of the most heavily tested populations [33]. The court cases covered in this article highlight the complicated relationship between intelligence and education vis a vis IDEA. More specifically, the psychometric properties of intelligence tests, historical conflicts surrounding racial and socioeconomic equity, differences in federal versus state policies, and methodological concerns surrounding special education research point to the difficulties of developing an educational system that can be perceived as equitable to and by all groups. Some of these complexities include: 1) Are intelligence measures that are standardized on a generalizable sample appropriate in all educational contexts, where sociodemographic factors can vary widely?, 2) What is the appropriate level of minority representation in IDEA given the documented group differences in intelligence and other standardized measures?, 3) How can the efficacy of IDEA be accurately assessed given services cannot be randomly assigned?, and 4) What measurable outcomes can determine the efficacy of IDEA and should these outcomes differ based on individual and group characteristics? While these questions will not eliminate all the controversies surrounding intelligence in education, they may provide a useful framework to allow future researchers, practitioners, administrators, lawyers, and policymakers to communicate and to collaborate with greater consistency and efficiency. 
